# Supplementary material for: Reconciling Mining with the Conservation of Cave Biodiversity: A Quantitative Baseline to Help Establish Conservation Priorities
Source: PLoS One. 2016 Dec 20;11(12):e0168348. doi: 10.1371/journal.pone.0168348 (PMC5173368; doi:10.1371/journal.pone.0168348)
Supplement: S1 Dataset — (ZIP) [file pone.0168348.s002.zip › Taxa/Serra Norte/SN_2013/N5S/Lista N5S-13.pdf]

CAVIDADE N5S-0013

Classe	Ordem	Fam/Outros	Gên/Outros	Espécie	S-U	U-S	Zon.
Arachnida	Acari	Asternoseiidae		sp.84	X		P
Arachnida	Acari	Astigmata		sp.3	X		P
Arachnida	Acari	Bdellidae		sp.61	X		A
Arachnida	Amblypygi	Phrynidae	*Heterophrynus*	*longicornis*	6	X	
Arachnida	Araneae	Araneidae	*Alpaida*	aff. *negro*	X		E
Arachnida	Araneae	Barychelidae		jovem	1		E
Arachnida	Araneae	Caponiidae		sp.1	X		A
Arachnida	Araneae	Ctenidae		jovem	1		E
Arachnida	Araneae	Filistatidae		sp.1	X		P
Arachnida	Araneae	Linyphiidae	*Meioneta*	sp.2	X		E
Arachnida	Araneae	Pholcidae		jovem	X		E
Arachnida	Araneae	Salticidae		sp.7	X		E
Arachnida	Araneae	Theraphosidae		jovem	2		P
Arachnida	Araneae	Theridiidae	*Theridion*	sp.1	X		E
Arachnida	Araneae	Theridiosomatidae	*Plato*	sp.1	X	X	E/P
Arachnida	Opiliones	Cosmetidae	*Roquettea*	*singularis*	1		E
Arachnida	Opiliones	Escadabiidae		sp.1		X	
Arachnida	Pseudoscorpiones	Olpiidae		sp.1	X	X	P
Chilopoda	Scolopendromorpha	Scolopocryptopidae	*Newportia*	sp.1	2		E
Diplopoda	Polydesmida	Pyrgodesmidae		sp.1	X	X	A
Entognatha	Diplura	Campodeidae		sp.1	X		E
Insecta	Blattodea	Blattidae		sp.5	9		P
Insecta	Blattodea	Polyphagidae		sp.1	15		P
Insecta	Blattodea			jovem	6	X	P
Insecta	Coleoptera	Chrysomelidae		sp.4	X		P
Insecta	Coleoptera	Elateridae		sp.1	X		P
Insecta	Coleoptera	Meloidae		sp.1	X		E
Insecta	Diptera	Cecidomyiidae		sp.		X	
Insecta	Diptera	Culicidae	*Anopheles*	*triannulatus*		X	
Insecta	Diptera	Culicidae	*Culex*	sp.		X	
Insecta	Diptera	Psychodidae	*Pifanomyia*	*gruta*	X	X	
Insecta	Heteroptera	Reduviidae	Emesinae	sp.		X	
Insecta	Heteroptera	Reduviidae	Reduviinae	sp.	1	X	P
Insecta	Homoptera	Auchenorrhyncha		sp.		X	
Insecta	Homoptera	Cixiidae		sp.5	X	X	E
Insecta	Hymenoptera	Formicidae	*Pachycondyla*	sp.1	X		E
Insecta	Hymenoptera	Formicidae	*Pheidole*	sp.		X	
Insecta	Isoptera	Rhinotermitidae	*Heterotermes*	sp.	X		P
Insecta	Isoptera	Termitidae	*Nasutitermes*	sp.	X	X	E
Insecta	Lepidoptera	Gelechioidea		sp.	X		E/P
Insecta	Lepidoptera	Noctuoidea		sp.1	1		P
Insecta	Lepidoptera	Tineoidea		sp.1	X	X	P
Insecta	Orthoptera	Phalangopsidae	*Paraclodes*	sp.	5	X	P
Insecta	Orthoptera	Phalangopsidae	*Phalangopsis*	sp.	18	X	P
Insecta	Psocoptera	Epipsocidae	*Epipsocus*	sp.2	X		E
Gastropoda	Pulmonata	Systrophiidae	*Happia*	sp.		X	
Malacostraca	Isopoda	Armadillidae		sp.1		X	
